# A Personalized Spring Network Representation of Emphysematous Lungs From CT Images

**DOI:** 10.3389/fnetp.2022.828157

**Published:** 2022-03-18

**Authors:** Ziwen Yuan, Jacob Herrmann, Samhita Murthy, Kevin Peters, Sarah E. Gerard, Hadi T. Nia, Kenneth R. Lutchen, Béla Suki

**Affiliations:** ^1^ Department of Biomedical Engineering, Boston University, Boston, MA, United States; ^2^ Department of Radiology, University of Iowa, Iowa City, IA, United States

**Keywords:** low attenuation area, clusters, mechanical failure, average symmetric surface distance, emphysema

## Abstract

Emphysema is a progressive disease characterized by irreversible tissue destruction and airspace enlargement, which manifest as low attenuation area (LAA) on CT images. Previous studies have shown that inflammation, protease imbalance, extracellular matrix remodeling and mechanical forces collectively influence the progression of emphysema. Elastic spring network models incorporating force-based mechanical failure have been applied to investigate the pathogenesis and progression of emphysema. However, these models were general without considering the patient-specific information on lung structure available in CT images. The aim of this work was to develop a novel approach that provides an optimal spring network representation of emphysematous lungs based on the apparent density in CT images, allowing the construction of personalized networks. The proposed method takes into account the size and curvature of LAA clusters on the CT images that correspond to a pre-stressed condition of the lung as opposed to a naïve method that excludes the effects of pre-stress. The main findings of this study are that networks constructed by the new method 1) better preserve LAA cluster sizes and their distribution than the naïve method; and 2) predict different course of emphysema progression compared to the naïve method. We conclude that our new method has the potential to predict patient-specific emphysema progression which needs verification using clinical data.

## Introduction

Emphysema is an important component of chronic obstructive pulmonary disease (COPD) characterized by flow limitation and increased lung compliance as a result of gradual destruction of the small airways and alveolar walls leading to the enlargement of peripheral airspaces (Snider, 1985; Snider, 1989). The risk factors for COPD include cigarette smoking, air pollution and genetic factors (Barnes, 2000). Decades of studies have revealed that various cellular and molecular mechanisms (Barnes, 2000; Barnes et al., 2003; Barnes, 2014) contribute to the pathogenesis and progression of emphysema including overexpression of proteases (Janoff, 1985; Churg and Wright, 2005), inflammation (Retamales et al., 2001; Churg et al., 2002; Turato et al., 2002), and extracellular matrix remodeling (Fukuda et al., 1989; Finlay et al., 1996; Vlahovic et al., 1999). Interestingly, alveolar wall rupture during mechanical stretching was also found to occur (Kononov et al., 2001), which has led to the proposition that mechanical forces in general play a key role in the progressive nature of the disease (Suki et al., 2003; Suki et al., 2013).

The severity of emphysema and in general COPD is evident via changes in the density on computed tomographic (CT) images (Goddard et al., 1982; Bergin et al., 1986) and quantified by the amount of low attenuation areas (LAA) (Gelb et al., 1993; Mishima et al., 1997). A clinical manifestation of disease progression is the yearly increase in the percent LAA (%LAA) (Yuan et al., 2009) defined by the ratio of total LAA and the lung area. A primary mechanism responsible for the progressive increase in %LAA is the mechanical force-induced rupture of alveolar walls (Mishima et al., 1999), enabled by the mechanical failure of fibers within the septal wall (Kononov et al., 2001). The underlying reason for this multiscale failure mechanism is that there is a pre-existing stress, or prestress, within the lung due to the transpulmonary pressure that distends the lung (Suki et al., 2011). The fibers are weakened by enzymatic digestion and abnormal matrix remodeling as a result of the complex inflammatory process in the tissue (Ito et al., 2005). Physiological levels of mechanical forces on fibers and septal walls are capable of rupturing them as the structure weakens. The prediction from this is that progression should be stronger in the apex where mechanical stresses are higher due to the weight of the lung. Indeed, as early as the 1971, an upper lobe predominance of emphysema was found to be associated with the topographical distribution of mechanical stress in the lung due to its own weight (West, 1971). More recent clinical observations found an accelerated rate of lung tissue deterioration after Lung Volume Reduction Surgery (LVRS) (Gelb et al., 1999; Gelb et al., 2001) that could be accounted for by mechanical force-enhanced deterioration at the apex of the lung predicted by an elastic network under the influence of gravity (Mondoñedo and Suki, 2017).

To quantitatively characterize the emphysematous lung structure, the distributions of contiguous LAA clusters were evaluated in a population of COPD patients, and the results showed that these distributions followed a power law with an exponent D that was closely associated with disease severity (Mishima et al., 1999). To interpret these findings, a spring network model was also used to mimic tissue deterioration. The network model predicted a decrease in D with advancing emphysema because the rupture of springs connecting adjacent LAA clusters led to the coalescence of neighboring clusters eliminating small and creating large clusters and hence effectively decreasing D (Mishima et al., 1999). The spring network model simulation thus suggested that the progression of emphysema cannot be solely due to enzymatic activity; instead, the enzymatic digestion and remodeling in tandem with redistributed mechanical forces increases the risk of failure of nearby structures giving rise to larger LAA clusters. Many subsequent studies have utilized the spring network approach to advance our general understanding of emphysema progression (Suki et al., 2003; Ingenito et al., 2005; Takahashi et al., 2014; Mondoñedo and Suki, 2017). However, the clinical utility of the network approach has remained limited because the network models have not considered the patient-specific details of the lung structure.

The aim of this study was to develop a method of constructing a spring network representation of the emphysematous lung based on clinical CT images thus leading to a capacity to perform personalized predictions of disease progression. To this end, we introduce a novel image processing technique to manipulate binary objects, which allows us to map LAA clusters of lung CT scans of emphysematous patients to spring networks. Our results show that the method preserves the structural characteristics of LAA clusters significantly better than a naïve method that simply maps LAA onto the elastic networks. Furthermore, we find that the new method predicts significantly different patterns of tissue deterioration than the naïve method, suggesting the potential for prediction of realistic patient-specific disease progression.

## Methods

A total of 20 slices from lung CT scans acquired at total lung capacity in 10 patients were selected from the National Lung Screening Trial dataset (National Lung Screening Trial ResearchAberle et al., 2011), such that the %LAA in each slice ranged from 5 to 40% of the total lung area segmented automatically (Gerard et al., 2020; Gerard et al., 2021). A pixel was considered LAA if its CT intensity was lower than −960 Hounsfield units, which is often used as a threshold for density-based quantification of emphysema (Tanabe et al., 2012). Axial 2D lung slices were mapped onto hexagonal linearly elastic networks of identical springs with unit spring constants. The springs in the network were prestressed, i.e., the distance between two nodes was larger than the resting length of the spring. A set of contiguous LAA pixels with 4-connectivity formed a LAA cluster. For each LAA cluster a mask was created in one of two ways (see below), which was then mapped onto the spring network. The springs that were inside the mask were eliminated from the network to mimic the low elastic recoil associated with emphysematous lesions. In addition, springs in high attenuation areas (defined as pixels with Hounsfield unit >−500) corresponding to for example blood vessels were assigned a resting length twice as large as regular springs. This ensured that these springs did not contract after optimization. The springs along the boundary of the lung area were fixed whereas those inside the lung area were free to move. The equilibrium configuration of the network was obtained by allowing the internal nodes of the network to move until the total elastic energy of the network was minimized using simulated annealing (Cavalcante et al., 2005). The spring networks were then converted to apparent CT images by assigning an intensity value to each pixel that is proportional to the mean spring stiffness within the area of the pixel as described previously (Wellman et al., 2018).

The first method was the naïve method (NM), in which the LAA cluster mask image was simply superimposed on the spring network and springs inside the mask were eliminated, creating void areas. However, eliminating springs led to an expansion of the holes after the equilibrium configuration of the network was obtained, which resulted in larger and more round shapes compared to the original LAA masks. To correct for the size and shape changes, we introduced a novel method, called the Curvature and Size Adjusted Method (CSAM), which preprocessed the LAA masks as follows. The preprocessing incorporated adjustment both in size and shape by manipulating the boundary of the LAA mask. The boundary pixels can be expressed as a closed curve on the *x-y* plane:

r(t)=(X(t),Y(t)) (1)

where r(t) is a parametric vector that points to the boundary of a LAA mask, X(t)  and Y(t) are the x and y coordinates of r(t) along two orthogonal base vectors, *t* is a parameter between 0 and 1 that describes the relative position along the boundary curve with respect to an arbitrary starting point at *t* = 0. To reduce the size of a LAA cluster, we resize the boundary as:

$$r_{s}\left(t\right)=\sqrt{\alpha }\cdot r\left(t\right)+\left(1-\sqrt{\alpha }\right)\cdot \overset{1}{\underset{t=0\;}{\int }}r\left(t\right)dt$$ (2)

where α is the ratio of the corrected LAA size to the original size. Note that when α=1 , $r_{s}\left(t\right)=\;r\left(t\right)$ . Alternatively, when α=0 , $r_{s}\left(t\right)=\overset{1}{\underset{t=0\;}{\int }}r\left(t\right)dt=constant$ and hence the boundary becomes a single point, the centroid of the original mask.

To correct for the changes in shape of a LAA mask, we first calculate the curvature along the boundary, decrease the curvature everywhere by a constant, and then reconstruct the LAA mask from the adjusted curvature profile. The curvature is calculated along the size-adjusted curve as

$$k_{s}\left(t\right)=\frac{\left|\left|T^{\prime }\left(t\right)\right|\right|}{\left|\left|r_{s}\prime \left(t\right)\right|\right|}$$ (3)

where T(t) is the unit tangent vector of $r_{s}\left(t\right)$ and the ′ denotes differentiation with respect to t . Here, $r_{s}\prime \left(t\right)$ can be calculated with either forward or backward difference approximation defined as:

$$r_{s}\prime^{+}\left(t\right)\;=\;\frac{r_{s}\left(t+\text{Δ}\right)-r_{s}\left(t\right)}{\text{Δ}}\;$$ (4)

and

$$r_{s}\prime^{-}\left(t\right)=\;\frac{r_{s}\left(t\right)-r_{s}\left(t-\text{Δ}\right)}{\text{Δ}}\;$$ (5)

where Δ≪1 .

$T^{'}\left(t\right)$ is then obtained using a 2^nd^ order central difference approximation:

$$\overset{\prime }{T}\prime \left(t\right)=\frac{r_{s}\prime^{+}\left(t\right)-r_{s}\prime^{-}\left(t\right)}{\text{Δ}}$$ (6)

A constant *β* is then subtracted from k(t) to obtain the curvature-adjusted $k_{cs}\left(t\right)=k_{s}\left(t\right)-\beta$ . Note that $k_{cs}$ is applied to the size-adjusted curve. To reconstruct $X_{cs}\left(t\right)$ and $Y_{cs}\left(t\right)$ from $k_{cs}\left(t\right)$ , we integrate twice with respect to t :

$$\theta \left(t\right)+\theta_{0}=\int k_{cs}\left(t\right)dt$$ (7)

$$X_{cs}\left(t\right)=\;-\int \cos\left(\theta \left(t\right)\right)dt$$ (8)

$$Y_{cs}\left(t\right)=\;\int \sin\left(\theta \left(t\right)\right)dt$$ (9)

Note that $\theta_{0}$ can be determined by brute force, allowing the reconstructed boundary to have the same rotational orientation as the original boundary.

The optimal values of α and β for LAA clusters of various sizes were determined by computational experiments mapping synthetic LAA clusters onto a spring network. Specifically, synthetic LAA maps, which were binary images of a single LAA in 4 different shapes, were resized to 6 different sizes containing 200 to 5,000 pixels. These synthetic LAA images were mapped onto spring networks using CSAM with α ranging from 0.5 to 1.0 and β from 0.0 to 0.3, both in steps of 0.02. The spring networks were then converted first to virtual CT images as described previously (Wellman et al., 2018) and thresholded to obtain the corresponding LAA maps. The error between the mapped LAA and the original LAA image was quantified by the average symmetric surface distance (ASSD, Supplementary Figure S1), which is a common metric to evaluate segmentation quality in CT and MRI research (Akkus et al., 2017). The main advantage of ASSD over other metrics, such as the total area overlap, is that ASSD is sensitive to dissimilarities in shape, not just size. An error map was obtained for each synthetic LAA image by assembling the error for all combinations of *α* and *β*. The optimal values of *α* and *β* were then determined for each error map by finding the region with the lowest error.

To further analyze the performance of NM and the CSAM, two elastic spring networks were constructed from each thresholded CT image resulting in a total of 40 networks. For each network, the configuration with lowest elastic energy was found as above. To simulate the progression of emphysema, the spring networks underwent progressive degradation by removing all springs that carried a force higher than 80% of the maximum force (Mishima et al., 1999). The new configuration corresponding to the minimal elastic energy was found and the procedure was repeated 10 times. At each iteration, the apparent CT images were used to quantify the changes in LAA structure during simulated disease progression. Structural parameters, including %LAA, the size of the largest LAA cluster and the average local difference between NM and CSAM were assessed at each iteration.

To evaluate the quality of LAA reconstructed in a spring network compared to the CT image of ground truth, 4 structural parameters of a lung CT or spring network-based apparent CT image were examined. These include the %LAA, the exponent S of LAA size distribution, the local difference of LAA clusters and the largest LAA cluster ( $A_{L}$ ). The $A_{L}$ cluster was obtained by counting the number of pixels of the largest LAA cluster of a lung CT slice. The local difference ( $D_{local}$ ) was quantified between the NM- and CSAM-generated apparent CT images. The apparent images were partitioned into grids of 10 × 10 squares, and the %LAA of each square was calculated as %LAA_i_. An example is shown in Supplementary Figure S2. The local difference was defined as the average absolute difference of $\%LAA_{i}$ between the apparent images:

$${\text{D}}_{\text{local}}=\frac{1}{N}\;\overset{N}{\underset{i=1}{\sum }}|\%{\text{LAA}}_{\text{i}}\left(\text{NM}\right)-\text{\%LA}A_{\text{i}}\left(\text{CSAM}\right)|$$ (10)

where N is the total number of squares in an apparent CT image, excluding those that contain no LAA clusters corresponding to both spring networks and NM and CSAM in the parentheses indicate which method was used to calculate the spring network-based %LAA. The quantification of %LAA was aimed at evaluating the quality of the reconstructed LAA cluster structure in the spring networks at the scope of the entire network. The ground truth, referred to as %LAA_GT_, was the %LAA of area occupied by LAA calculated for each CT slice. The difference between %LAA calculated from the spring networks and %LAA_GT_, referred to as *E*_*LAA*_, characterized the error produced by the reconstruction algorithm. The LAA size distributions in CT and the spring networks were analyzed on the log-log domain. Since the distributions show a region of linear decrease, the exponent D , the negative slope of regression line fit to cumulative distribution of LAA sizes, was used to characterize the distributions.

ANCOVA was applied to analyze the relationship between the values of optimal parameters and LAA sizes among the 4 groups of LAA shapes. Linear regression was used to quantify the dependency of *E*_*LAA*_ on %LAA_GT._ Linear regression was also applied to fit the change of *D*_*local*_ against the progression of disease. Wilcoxon Rank Sum test was used to compare the absolute values of *E*_*LAA*_ between the naïve and CSAM groups. Linear regression and Welch’s test was used to compare S in different groups. *p* < 0.05 was considered statistically significant for all analyses. All image and data analyses were performed with MATLAB.

## Results

**FIGURE 1:**
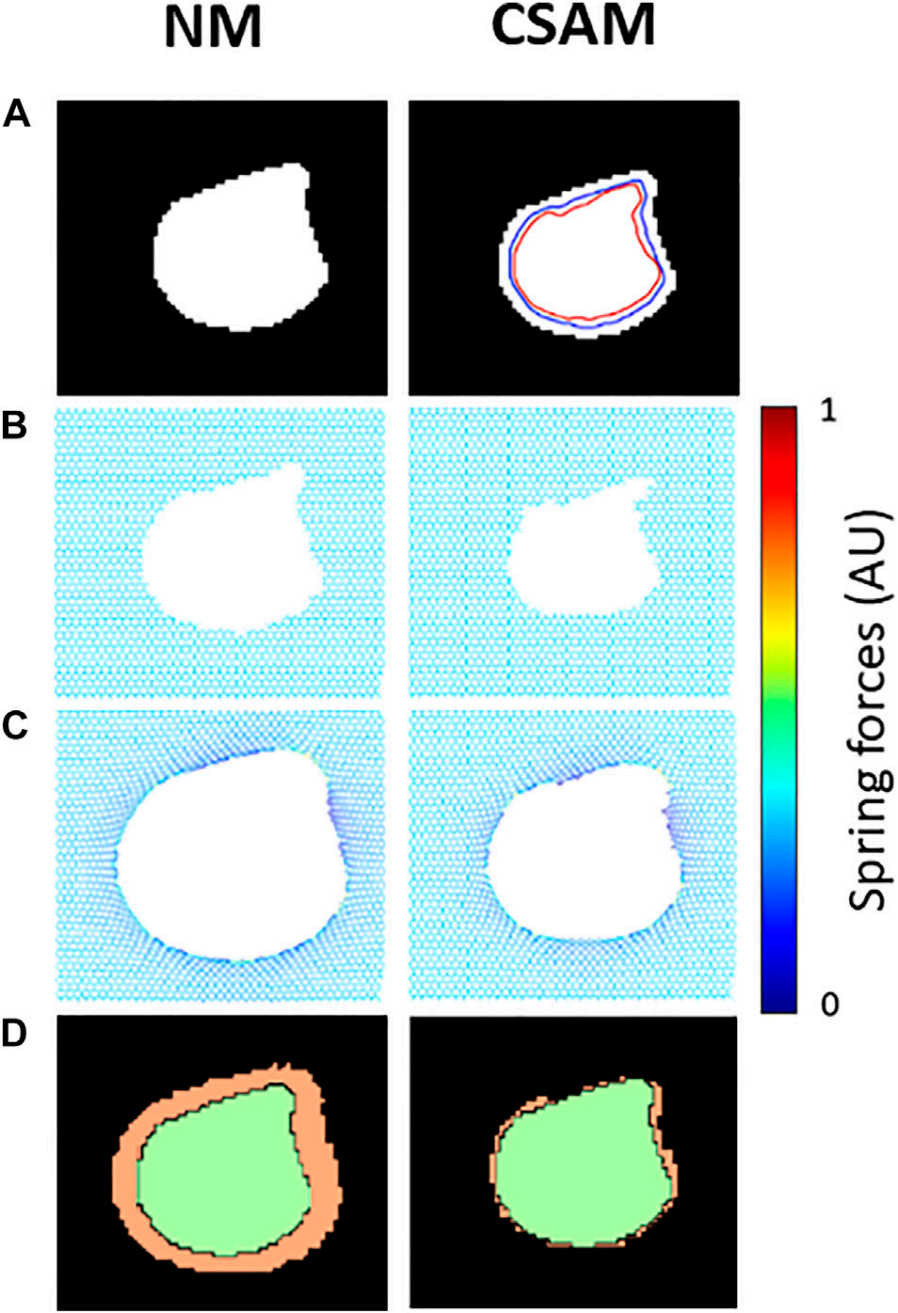
Examples of mapping a synthetic low attenuation area (LAA) cluster onto spring networks using the naïve method (NM) and the Curvature and Size Adjusted Method (CSAM). **(A)** Binary image of the LAA. Blue and red lines represent respectively the size and curvature steps of the CSAM. **(B)** Removal of springs in a prestressed elastic network. **(C)** Configuration of the networks after solving for mechanical equilibrium. Note that dark blue and yellow represent low, and high mechanical force on springs. **(D)** Comparison of the reconstructed LAAs (green) and the original LAA (orange).

**FIGURE 2:**
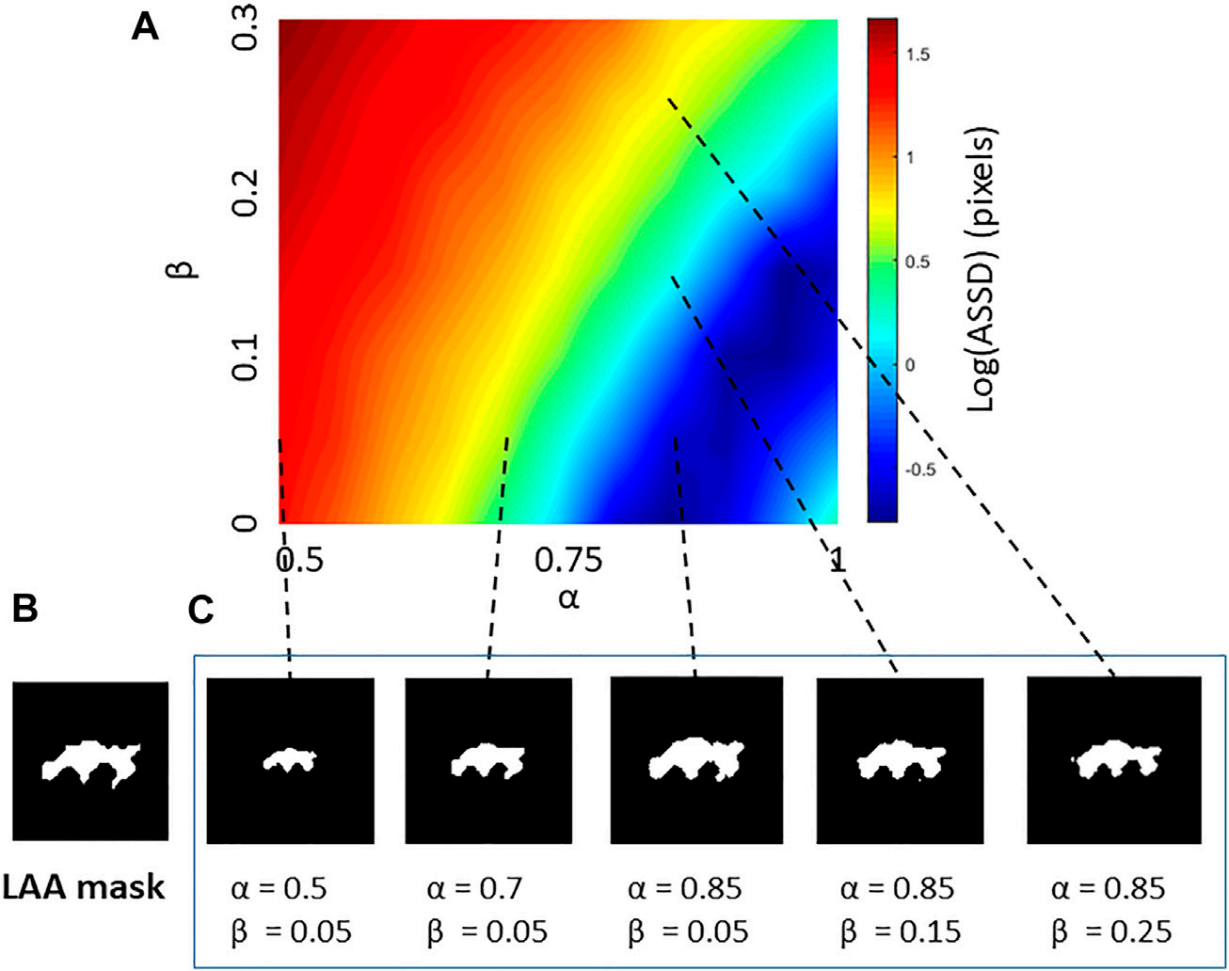
**(A)** An example error map characterizing the morphological differences between a low attenuation area (LAA) cluster from a CT image and LAA clusters reconstructed by the Curvature and Size Adjusted Method (CSAM). The error, increasing from blue to red in log scale, is given in terms of the average symmetric surface distance (ASSD) as a function of the size parameter *α* and the shape parameter *β*. **(B)** Target LAA cluster in CT image. **(C)** Reconstructed LAA clusters with different combination of parameters with dashed lines pointing to their error values on the map.

**FIGURE 3:**
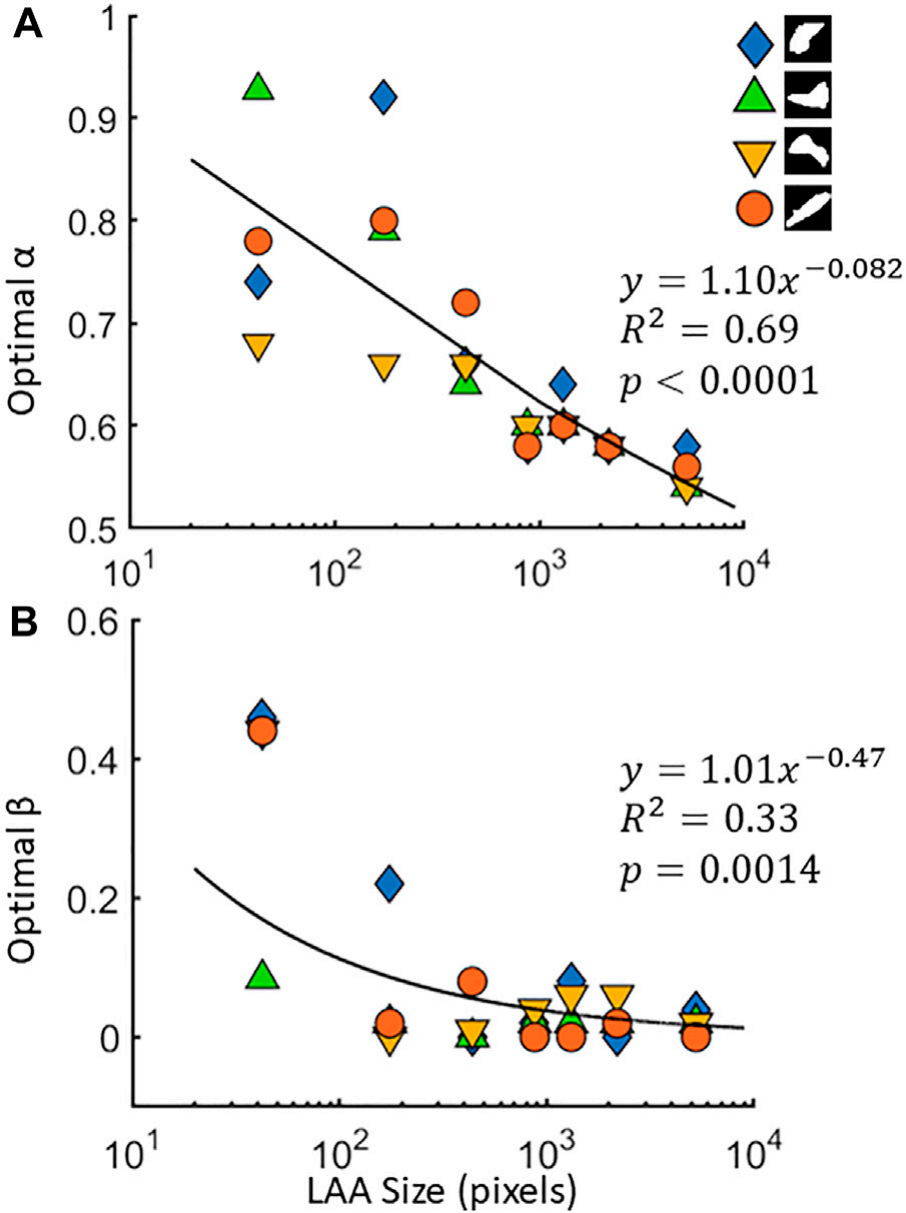
The optimal values of the size parameter *α* **(A)** and shape parameter *β* **(B)** for 4 different low attenuation area (LAA) cluster shapes. The target LAA clusters were resized to 7 different values across 3 orders of magnitude, and the optimal parameters are obtained for each size. Regressions for *α* and *β* are also shown.

An example of the performances of the NM and the CSAM is shown in Figure 1. It can be seen that CSAM better approximates the original LAA shape and size than the NM. In total, 24 artificial CT images were generated with LAAs in 4 random shapes and 6 different sizes. Every image was reconstructed with the spring network with all combinations of the size and shape parameters, α and β , respectively, to obtain error maps. The error between the original and the reconstructed LAAs was quantified with ASSD. An example error map is displayed in Figure 2. Errors corresponding to the NM are at the origin (0,0) of these maps. The optimal values of α and β corresponding to the lowest ASSD were then plotted for the 4 shapes as a function LAA cluster size in Figure 3. The optimal parameters associated with the lowest errors of each map were analyzed with ANCOVA, which revealed that LAA size had a statistically significant effect on the optimal value of *α* (*p* < 0.001) but LAA shape did not influence α (*p* = 0.37). Interestingly, neither size nor shape had a statistically significant effect on the optimal value of *β* (*p* = 0.46 and *p* = 0.44, respectively). Furthermore, a weak power law relationships were used to describe the dependence of optimal parameters on LAA size (see Figure 3). The power law relationship was used to select the optimal α for the spring network-based reconstruction of CT images that contain thousands of LAA clusters of different size and shape.

**FIGURE 4:**
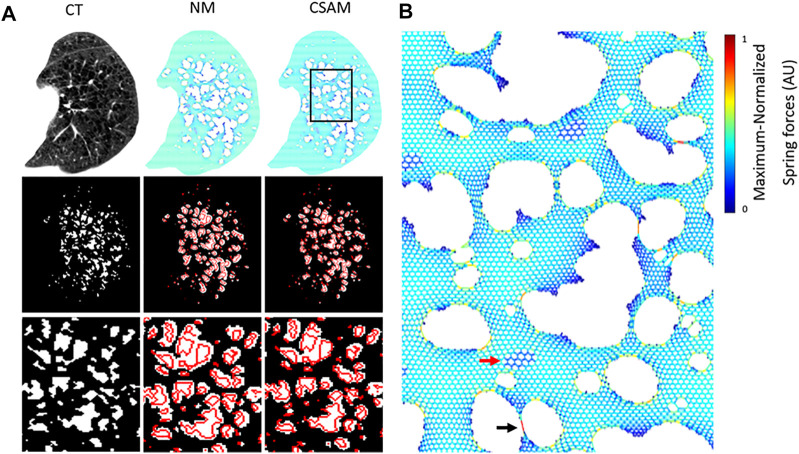
**(A)** Original CT from a patient and examples of mapping to spring networks using the naïve method (NM) and the Curvature and Size Adjusted Method (CSAM). The left column displays the CT scan of an emphysema patient (top), the binary low attenuation area (LAA) map obtained by thresholding at −960 Hounsfield Unit (middle), and a zoomed-in region of the LAA map (bottom). The middle and right columns represent the same content for two spring networks constructed from the CT image with the NM and CSAM. The red lines represent the boundaries of LAA clusters on the original CT. **(B)** Zoomed-in version of the rectangle on the CSAM network in **(A)**. Colors are proportional to force. The black arrow points to narrow bridge carrying a high force whereas the red arrow marks a region of high attenuation.

Spring networks were constructed from CT images from the National Lung Screening Trial (NLST) dataset using both the NM and CSAM with optimal parameters. The target CT images were selected so that the LAA% ranged from a low value of ∼3% (early emphysema) to a value >40% (advanced emphysema). Figure 4A displays a representative CT image, the corresponding binary LAA structure as well we the spring networks processed with NM or CSAM and the corresponding reconstructed LAA images. The binary images in the third row are zoomed-in regions of interest emphasizing a large LAA cluster with red lines on the reconstructed images representing the edges of original LAA clusters. Visual assessment suggests that compared to NM, the CSAM approximated considerably better the original LAA clusters. Figure 4B shows another example of the spring network obtained with CSAM. A magnified region in panel C demonstrates the distribution of mechanical forces represented by the colors.

**FIGURE 5:**
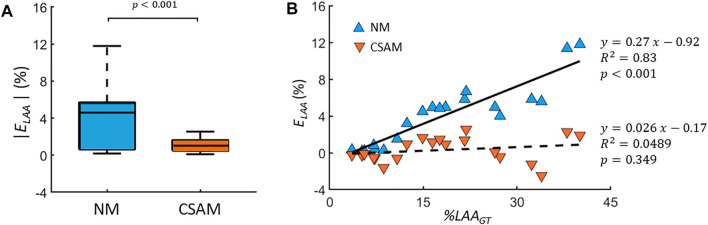
**(A)** Absolute error (*E*_*LAA*_) between the percent low attenuation areas (%LAA) of CT scans and reconstructed images using either the naïve method (NM) or the Curvature and Size Adjusted Method (CSAM). The graphs show the medians (lines), 75th percentiles (boxes) and 90th percentiles (error bars). **(B)** *E*_*LAA*_ values as a function %LAA of CT images (%LAA_GT_). Errors corresponding the NM and CSAM are shown with filled blue and red triangles, respectively, and the solid and dashed lines are the corresponding regression lines.

To quantify the difference between original CT images and reconstructed ones, the error *E*_*LAA*_ was calculated for 20 CT images with varying %LAA with both methods. As Figure 5A shows, the error corresponding to NM was ∼10 times larger than that of CSAM (4.10 ± 3.41 vs 0.32 ± 1.35, *p* < 0.001). Additionally, Figure 5B demonstrates that *E*_*LAA*_ increased linearly with %LAA_GT_ using the NM (*R* ^2^ = 0.83, *p* < 0.01), while *E*_*LAA*_ was not a function of %LAA_GT_ using the CSAM (*R* ^2^ = 0.0489, *p* = 0.349). Note that the negative values correspond to LAA clusters smaller than those on the original CT image. The correlation between *E*_*LAA*_ and %LAA_GT_ in the NM is a result of the prestress that deforms the network to a greater level as springs are removed to mimic the more advanced disease stages with larger %LAA. The lack of such correlation in the CSAM indicates that the preprocessing of LAAs successfully attenuated the influence of prestress in the network.

**FIGURE 6:**
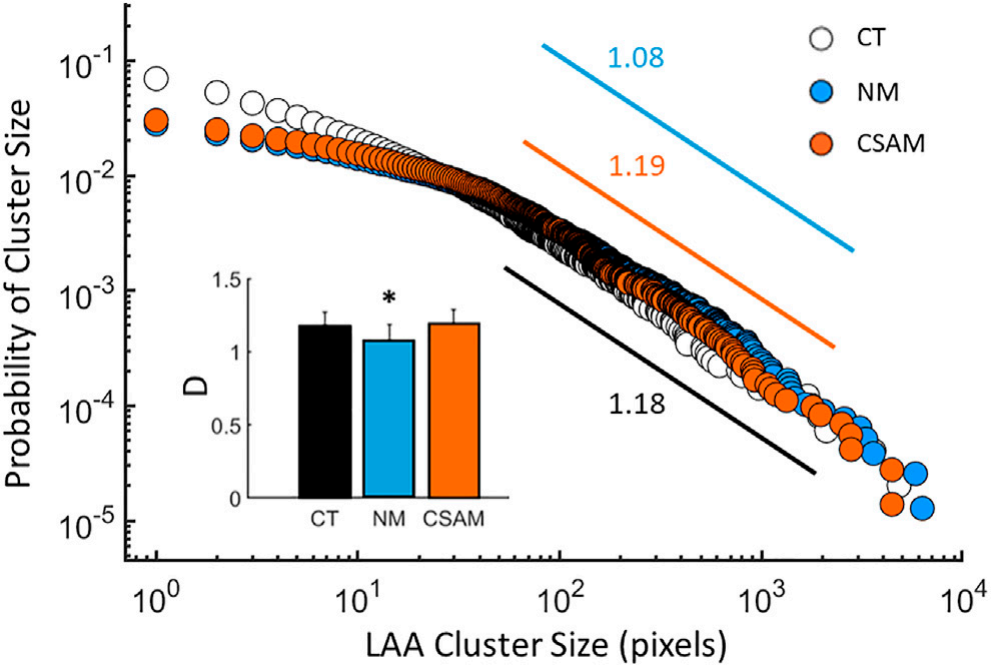
The distribution of low attenuation area (LAA) clusters computed from the CT images of 7 subjects (open circles) and reconstructed with the naïve method (NM, blue) and the Curvature and Size Adjusted Method (CSAM, red). The exponents, defined as the negative slopes, are given next to the regression lines between pixel values of 50 and 1,000. Inset: Mean and estimated standard deviation of the exponents of LAA cluster distributions. The * indicates p<0.05 compared to the CT value.

The size distribution of LAAs displayed a region of linear decrease on a log-log graph for the original CT images as well as the reconstructed images with both methods (Figure 6) consistent with a power law distribution of LAA sizes. The CSAM preserved the slope of regression line from size 50 to 1,000 pixels consistent with that obtained from CT scans. For LAAs with pixel size between 1 and 50, the CSAM distribution is almost identical to NM distribution. However, there was a small but statistically significant difference between the exponents of the distribution of the original CT and NM (*p* < 0.0001). In contrast, there was no difference between the exponents of the original CT and CSAM network-derived distributions (inset, Figure 6).

**FIGURE 7:**
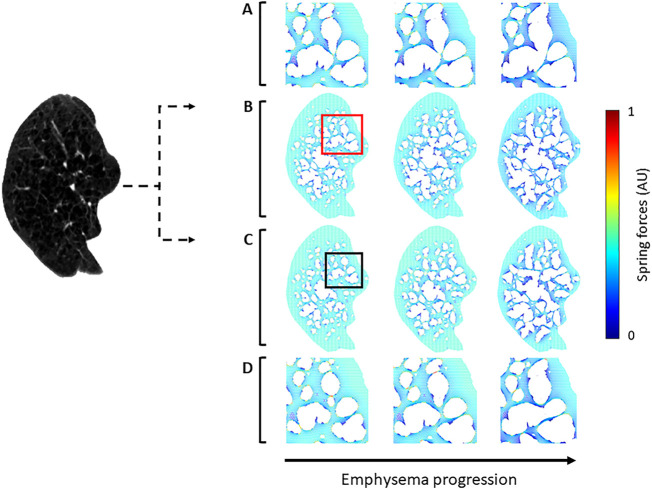
Two spring networks (right) reconstructed from a single CT image (left) using the naïve method (NM) and the Curvature and Size Adjusted Method (CSAM). The first column represents the reconstruction whereas the second and third columns show mechanical force-based network breakdown mimicking the progression of emphysema. **(A)** Magnified views of the red square region in row **(B)**. **(B)** spring network constructed with NM. **(C)** spring network reconstructed with CSAM and simulation of emphysema progression. **(D)** Magnified view of the black square region in row **(C)**.

To test whether the prediction of disease progression depends on the initial structural differences generated by the two methods, the progression of emphysema was simulated using both approaches. Networks created by NM and CSAM were used iteratively to mimic tissue degradation by eliminating springs bearing a force higher than 80% of the maximum. Figure 7 displays an example CT image and elastic network representations before and two steps after simulated emphysema progression. At the initial stage (the left column), the general morphology of LAA clusters represented by the voids in the networks looks similar for the NM and CSAM. However, the locations of the springs carrying the highest forces are different. As the degradation progressed, the structural differences increased between the two methods.

**FIGURE 8:**
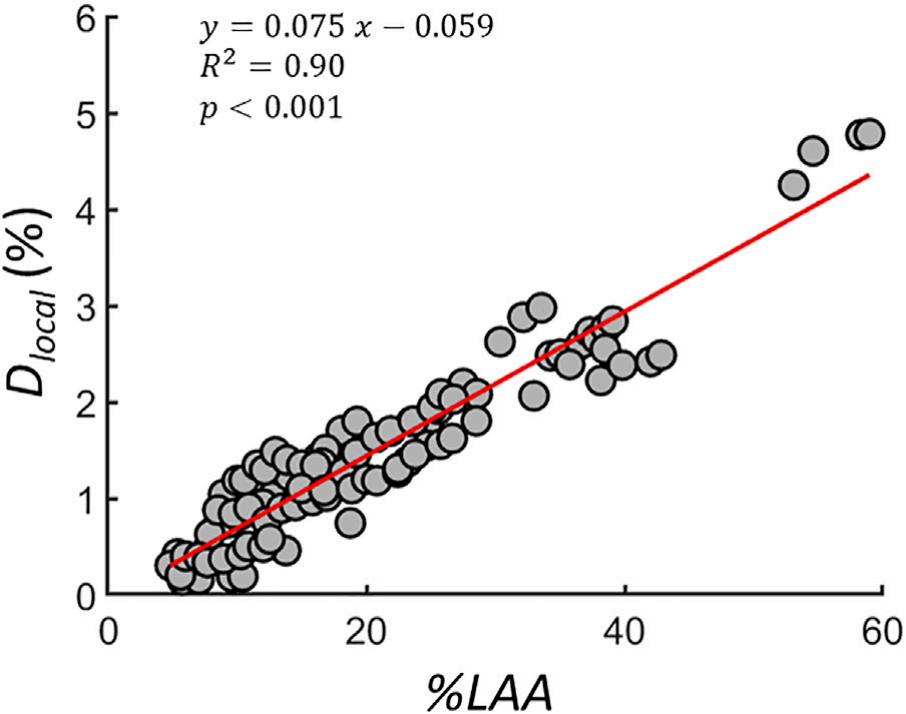
The percent local difference ( $D_{local}$ ) between two spring networks during iterative simulation network breakdown. $D_{local}$ is calculated according to Eq. 10 to quantify differences in LAA structure on apparent CT images constructed from the same CT scan with NM or CSAM.

**FIGURE 9:**
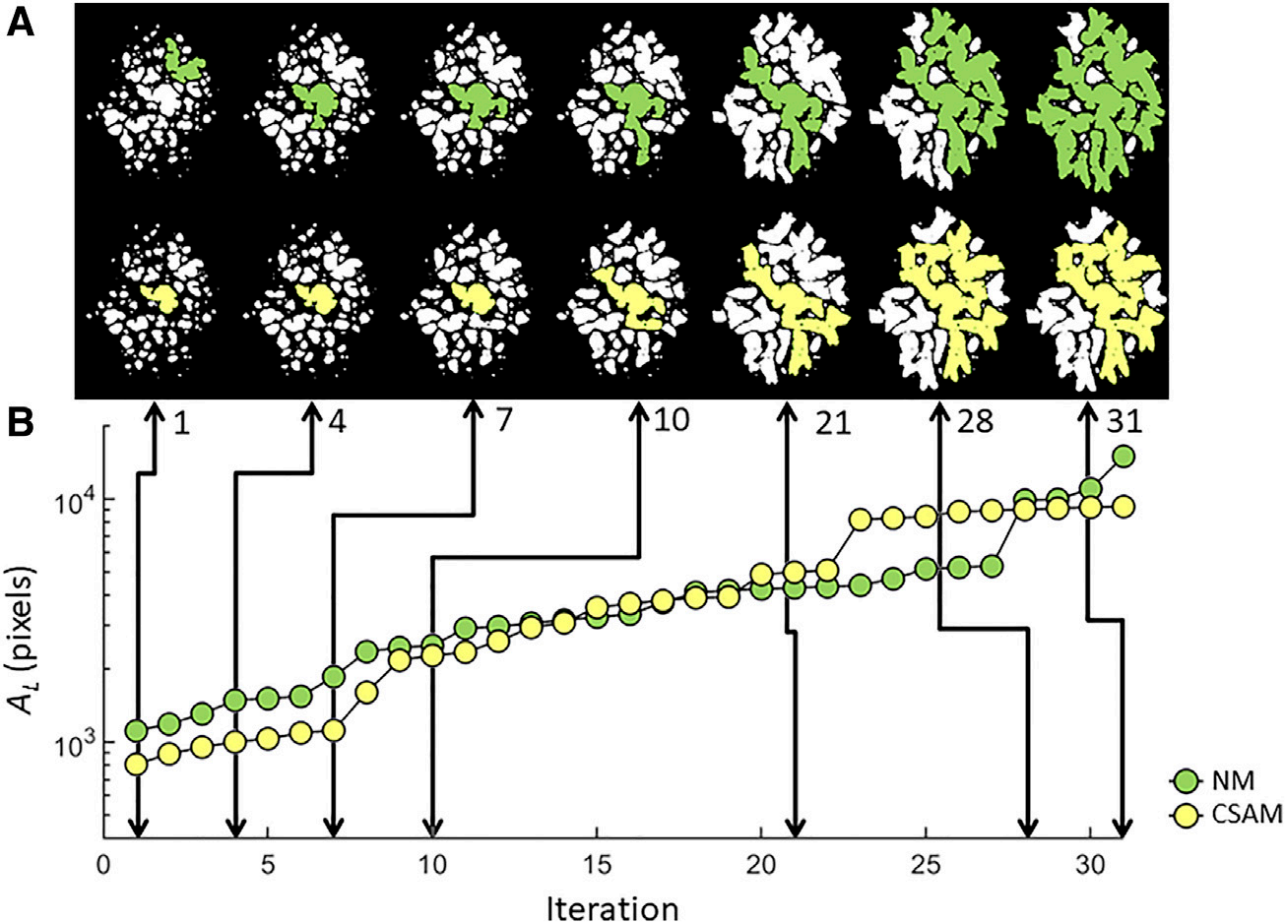
LAA configurations **(A)** and the evolution of the largest LAA cluster **(B)** traced through simulated network breakdown in two spring networks constructed with NM or CSAM using the same patient CT image. Note that even when the largest clusters have similar size (iterations 1, 10 and 20), their morphological difference is apparent.

To quantify the average local differences between the spring networks, *D*_*local*_ was calculated during the simulated progression. Springs were eliminated from the networks, which were converted to apparent CTs to calculate *D*_*local*_ at similar disease severity or total %LAA. Figure 8 shows the *D*_*local*_ of 10 subjects as a function of %LAA . *D*_*local*_ was significantly associated with disease severity ( $R^{2}=0.9,\;\;p<0.001$ ), indicating that LAA clusters in the spring networks constructed with CSAM evolved with a different pattern. Furthermore, the increase of the local difference was invariant of initial %LAA since the linear relationship included CT images of 10 subjects with distinctly different LAA structure and %LAA. Lastly, the evolution of the largest LAA cluster was traced for one subject through 30 iterations in Figure 9. There are sudden jumps in cluster size implying the coalescence of two neighboring clusters. Interestingly, the phenomenon of coalescence takes place at different iteration numbers for the two mapping methods. At the end of the iterations, the largest cluster size produced by the NM substantially exceeded that produced by the CSAM.

## Discussion

In this study, we introduced a new method for constructing elastic spring network representations of emphysematous lungs based on patient CT images to enable personalization of simulated emphysema progression. The main findings are as follows: 1) CSAM produced spring networks that better preserved the total area of LAA clusters as well as their size distributions compared to the NM; and 2) the structure and the rate of emphysema progression predicted by the CSAM are different from those produced by the NM.

The airways, alveolar ducts, and alveoli are maintained distended in the lung by the prestress due to the transpulmonary pressure generated by the negative pleural pressure in the thoracic cavity. This is mimicked in the network model by assigning spring rest lengths that were smaller than their length used during the mapping of CT images to the network. The mechanical properties of lung parenchyma are dominated by ECM and associated fibers (Suki et al., 2011), especially at high lung volumes where the CT images are taken. Hence collagen fibers due to their recruitment also provide non-linear parenchymal elasticity (Maksym and Bates, 1997; Jawde et al., 2021). In addition, airway mechanical properties are also known to behave nonlinearly (LaPrad et al., 2013; Eskandari et al., 2018). However, in this study we were not modeling inflation and deflation. Hence, tissue elasticity was represented by linear springs within the network. Additional simulations were carried out (Supplementary Figure S3) demonstrating that the evolution of the cluster structure depends on tissue nonlinearities, which tend to smooth the LAA cluster boundaries. However, the CSAM and the NM methods clearly lead to different evolutions of the largest LAA cluster.

The spring constants were set to unity despite the well-known parenchymal stiffness values reported previously using various techniques (Lai-Fook et al., 1976; Cavalcante et al., 2005; Maghsoudi-Ganjeh et al., 2021). The absolute value of the spring constants, however, does not influence network breakdown. What is apparently more important during emphysema progression is that alveolar septal walls and ducts are gradually destroyed through the mechanisms of rupture (Kononov et al., 2001), which was simulated by breaking the springs according to a force-based rule as previously (Mishima et al., 1999; Suki et al., 2003; Ingenito et al., 2005; Takahashi et al., 2014). This is a critical step since following rupture, there is a redistribution of mechanical forces which increases the likelihood of further rupture at locations of high stresses (Suki et al., 2003). To model this, we used a threshold of 80% of the maximum force. Simulations using 70 and 90% thresholds provided similar results (Supplementary Figure S4) to those in Figure 8 suggesting that the conclusions in this study are not sensitive to the value of the force threshold.

To model the tissue breakdown process in a personalized manner, we mapped LAA clusters from CT images onto the network model. The mapping is, however, not unique, and in this study, we investigated two methods. The first is the rigid NM, which simply creates holes in the network by removing all springs corresponding to each LAA cluster on a CT image. This simple method, however, does not consider the presence of prestress and hence it allows expansion of LAA regions after the network is solved for mechanical equilibrium, as demonstrated in Figure 1. In the ideal scenario when there is only a single LAA cluster on the CT image, the expansion of the LAA cluster is more enhanced for larger clusters since the error grows with %LAA (Figure 5B). To tackle this problem, the size parameter α in the CSAM attempts to correct for the expansion by first reducing the size of hole compared to the original LAA cluster on the CT image. The asymptotic behavior of α for large values of LAA size can also be explained by the constraints of the network boundary, where springs are not allowed to move, and prestress-induced expansion is therefore not as significant. As a result, the efficacy of α could be biased for LAA clusters located near the network boundary, which might also explain at least partially the error produced by the CSAM in Figure 5. It still important to note that the error following CSAM is not zero. Possible explanations for this include the boundary effect in the resizing step similar to NM, a filtering effect of the prestress that rounds sharp corners of LAAs and the inevitable error of converting spring networks to apparent CT images. Nevertheless, CSAM was able to reproduce the exponent of the LAA size distribution calculated from the CT images while NM produced a smaller exponent (inset, Figure 6).

Previous studies reported decreased exponents of the power law LAA distribution in COPD patients, COPD patients with smoke history and COPD patients with history of exacerbation compared to normal subjects, subjects without smoke history, and subjects without history of exacerbation (Tanabe et al., 2011; Tanabe et al., 2012). A decrease in the exponent was interpreted using a model in which coalescing neighboring LAA clusters is favored probabilistically (Tanabe et al., 2012). This model is equivalent to an elastic spring network in which springs are eliminated with a probability proportional to the force they carried (Mishima et al., 1999; Mondoñedo and Suki, 2017). Alternatively, using a CT image-based model, normal pixels were switched to emphysematous pixels with a probability inversely proportional to their distance to a large LAA cluster (Mondoñedo et al., 2019). This method also embodies mechanical forces since tissue regions that separate large LAA clusters carry a large force with a high probability.

Generally, the decreased exponent is consistent with a higher probability of finding larger clusters which results from the coalescence of LAA clusters where damaged alveolar wall ruptures under the effect of mechanical forces during disease progression. Indeed, the lower exponent corresponding to the NM is due to the expansion of LAA clusters, leading to decreased distances and higher mechanical forces between neighboring LAAs (Figure 7). The higher forces in turn accelerate network breakdown during simulated disease progression (Figure 9). The deviation between the CT and the CSAM and NM distributions for LAA sizes between 1 and 50 pixels is probably a resolution issue stemming from small clusters during either the mapping of LAAs to the spring network or reconstructing apparent CT images from the network. Taken together, CSAM provided small and LAA-size-independent error with a distribution of their sizes consistent with the original CT image. Hence, networks created by the CSAM have the potential to predict the evolution of disease in a personalized manner.

Although patient-specific spring networks for predicting emphysema progression have not been proposed, previous elastic networks were applied to study the structure-function relationships during disease progression or following lung volume reduction, a procedure used to reduce the severity of advanced emphysema (Cavalcante et al., 2005; Takahashi et al., 2014; Mondoñedo and Suki, 2017). Our current study utilizes patient-specific CT images not only to provide structural information, but also to predict spatial pattern of the evolution of LAA clusters during disease progression. We simulated the progression of emphysema as an iterative process degrading springs carrying the highest forces which incorporates the notion that local inflammation generates enzymatic damage which in turn reduces the failure stress and strain of collagen and elastin fibers and ultimately the septal wall (Ito et al., 2005). While many different implementations of failure models can be used, we are interested in the extent to which CSAM-generated networks predict different disease trajectories than NM-generated networks. The results demonstrate that the structural differences corresponding to the CSAM, and NM networks accumulate with iterations (Figure 9). The structural difference is quantified by *D*_*local*_ that captures both the spatial and morphological discrepancy of LAA clusters between the two networks. The larger the total %LAA, the larger the effect of prestress in expanding the clusters in the NM which results in an increase in by *D*_*local*_ with %LAA (Figure 8). Spatial differences and the growth of the largest LAA cluster suggest that the evolution of LAA cluster structure is highly sensitive to the initial network configuration. Indeed, the largest LAA cluster displays with distinct patterns with sudden jumps that result in a larger final largest cluster in the NM network. This is important since a recent study reported that in 3D, large LAA clusters coalesce and form a super cluster with probability approaching 1 in late-stage emphysema (Mondoñedo et al., 2019). Furthermore, these super clusters not only characterize emphysema, but appear to drive disease progression by changing mechanical forces around its boundaries creating at risk regions of emphysema in normal tissue.

The CSAM networks thus have the potential to become a clinically useful tool in predicting emphysema progression. Before using this approach, it should be experimentally validated. Verifying the predictions of the model can in principle be achieved by comparing them to repeated CT images obtained in COPD patients. Within the current data set, this is unfortunately difficult for several reasons. First, the total lung volume at which the CT images were taken in consecutive years is not the same, often resulting in a reduction during progression, which in turn can also generate a decrease in the total number of low attenuation voxels. Second, a successful prediction would require mapping the selected CT slice to the network, mimicking disease progression, and comparing the virtual image from the model to the same CT slice of the patient taken at a later time. However, finding the same slice can also be problematic if the lung volume is different, and the lung undergoes heterogeneous tissue destruction in 3D. One solution is to use 3D image registrations and extending the current method into 3D, both of which are beyond the scope of the present work.

The study was based on several simplifications resulting in the following limitations: 1) The lung was modeled by a homogeneous spring network even though there are large variations in tissue stiffness within the parenchyma (Liu and Tschumperlin, 2011) which appear to increase with aging (Sicard et al., 2018). Future models should include local variations in stiffness, which should also influence the pattern of network breakdown. 2) The effects of airways on parenchymal distension was ignored, despite recent data showing a significant association between airway obstruction and emphysema severity (Bhatt et al., 2016). Computational simulation also uncovered that airway contraction-induced force propagates farther in an elastic spring network model compared to in an elastic continuum model (Ma and Bates, 2012). Hence, a more realistic model should include the effects of airways especially because the airway walls are stiffer than the parenchyma (Sicard et al., 2018). 3) The size of the hexagons in the network was similar to the pixel size in the CT images. Thus, the network resolution is too coarse to incorporate all details needed for conversion between spring network and apparent CT images. A ratio of 2:1 between the dimensions of the CT pixel and a finite element representation was shown to limit the error of conversion between CT and network to a relatively small level (Diciotti et al., 2015). 4) 3D interactions were neglected as we used 2D models. The study that discovered the super clusters suggest that these large percolating structures emerge in 3D (Mondoñedo et al., 2019). Even though Figure 9 shows super clusters, this is not generally observed in 2D. Therefore, the current method should be extended into 3D for more relevant personalized predictions.

In summary, we introduced a novel method of constructing patient-specific spring network models based on 2D slices of lung CT scans. The new method is significantly better than a simple naïve method of mapping LAAs to the network. Despite the simplifications used, our novel mapping approach compensates for LAA size and shape alterations after solving for mechanical equilibrium under conditions representing prestressed lung tissue and has succeeded in preserving the structural characteristics and statistical properties of LAA clusters seen on patient CT images. Furthermore, we demonstrated that the simulated growth and coalescence of LAAs in spring network models is sensitive to the method of initial network generation. This method has the potential to improve patient-specific prediction of emphysema progression and therapeutic intervention, particularly when combined with a realistic representation of disease progression and airspace enlargement.

## Data Availability

The original contributions presented in the study are included in the article/Supplementary Material, further inquiries can be directed to the corresponding author.
