# Supplementary material for: A Personalized Spring Network Representation of Emphysematous Lungs From CT Images
Source: Front Netw Physiol. 2022 Mar 18;2:828157. doi: 10.3389/fnetp.2022.828157 (PMC10013051; doi:10.3389/fnetp.2022.828157)
Supplement: Supplementary file 1 [file DataSheet1.pdf]

A PERSONALIZED SPRING NETWORK REPRESENTATION OF EMPHYSEMATOUS LUNGS FROM CT IMAGES

Ziwen Yuan[1], Jacob Herrmann[1], Samhita Murthy[1], Kevin Peters[1], Sarah E. Gerard[2], Hadi T. Nia[1], Kenneth R. Lutchen[1] and Béla Suki[1]

[1]Department of Biomedical Engineering, Boston University, Boston, MA, USA

[2]Department of Radiology, University of Iowa, Iowa City, IA

Supplemental Figures

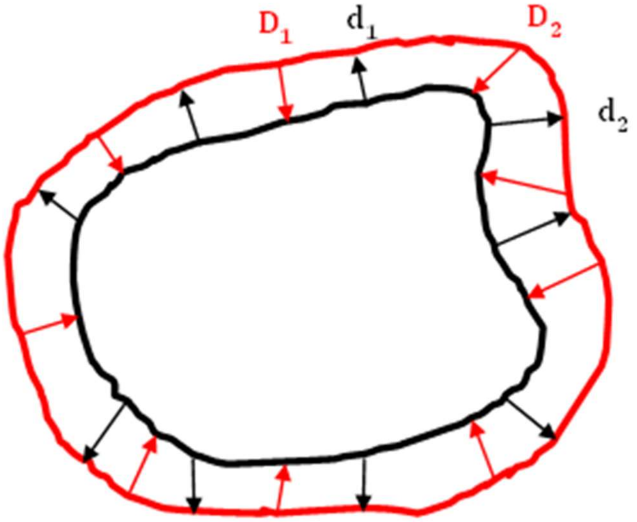

$$E_{ASSD} = \frac{1}{2N}\sum_{i=1}^{N} D_i + \sum_{j=1}^{N} d_j$$

Figure S1: Demonstration of the calculation of the average symmetric surface distance (ASSD).

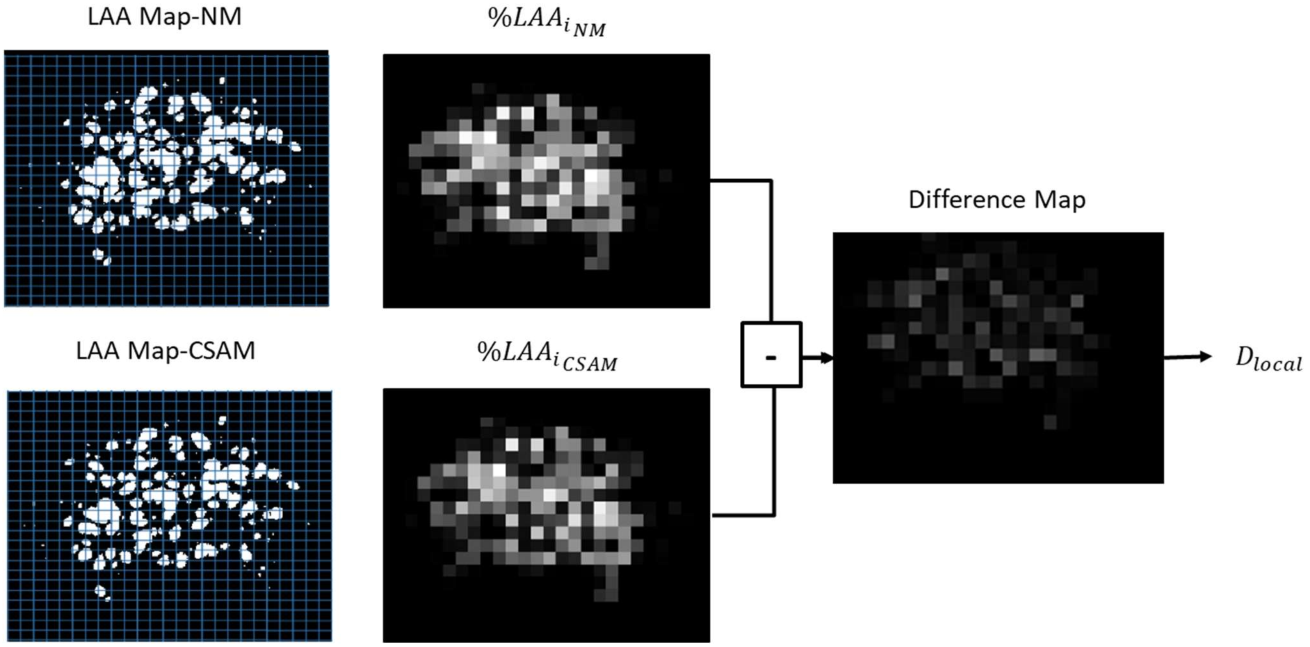

Figure S2: An example of how the local difference was calculated: 1) a grid was placed on the image of LAA clusters (left column), 2) the intensity of each grid element representing a pixel in the coarse-grained image was determined based on the number of white and black pixels (middle column), 3) a difference map was obtained (third column) and 4) the average absolute difference of $\%LAA_i$ between the apparent images was calculated according to Eq. 10 in the main text.

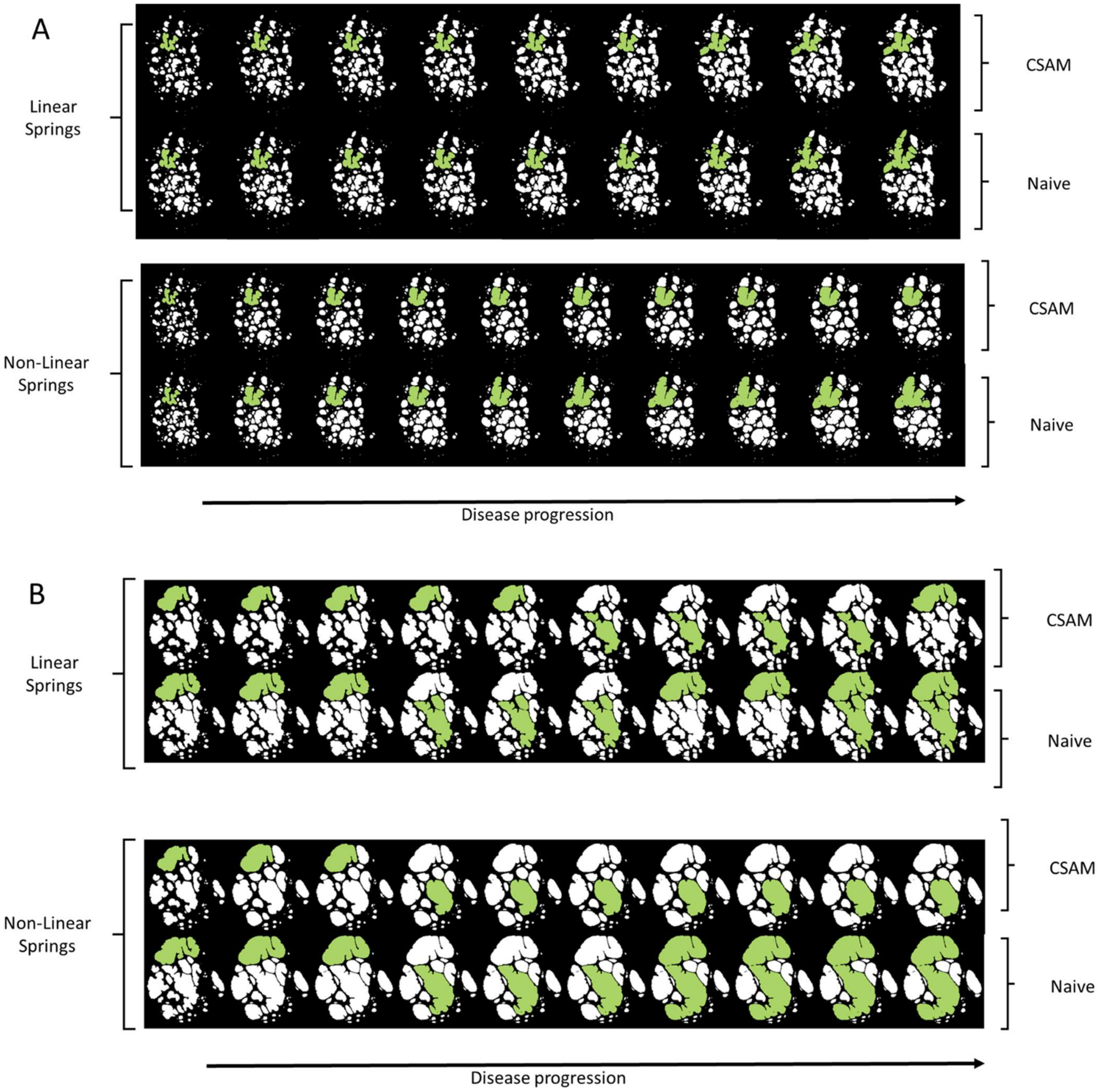

Figure S3: Simulated evolutions of LAA clusters during the progression of emphysema. The highlighted green regions represent the largest cluster in each network. Panels (A) and (B) correspond to 2 patients. The linear and non-linear networks start from the same configuration, displayed in the first columns. Identical criteria of disease progression were applied to all simulations (see main text). Linear spring constants were given a value of 1, whereas the second and third order spring constants were assigned values of 10 and 100, respectively.

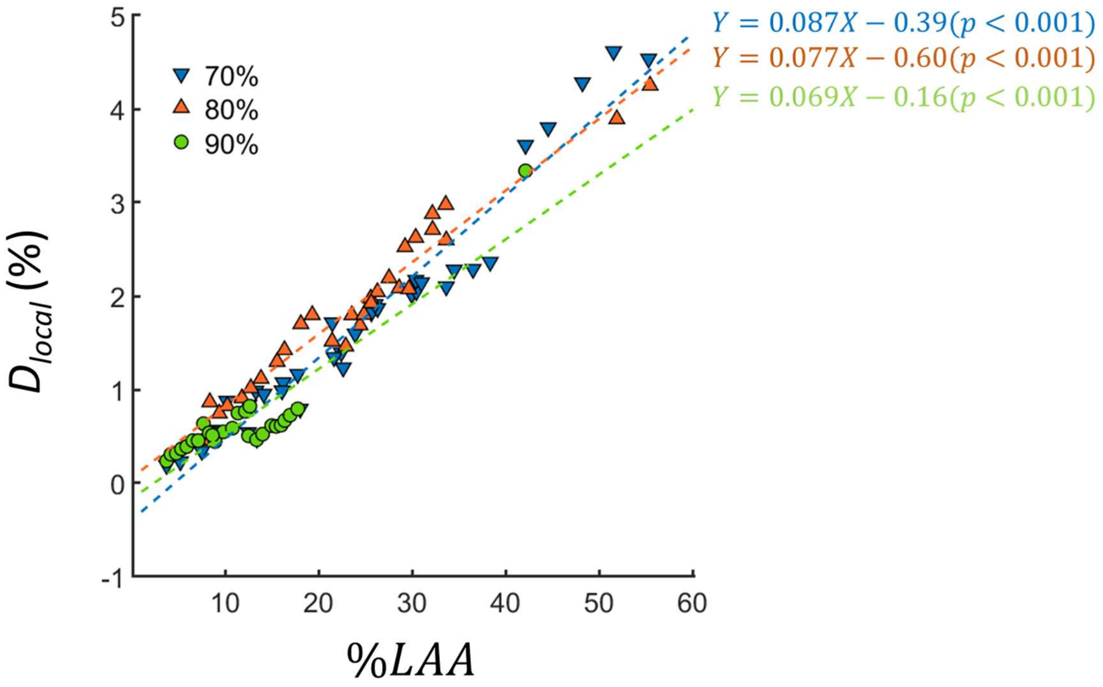

Figure S4: The relationship between the local difference D_{local} and *%LAA* for the NM and CSAM methods during the prediction of emphysema progression. Spring network models constructed with both NM and CSAM simulated the progression of emphysema through iterative degradation and the local difference was calculated between NM- and CSAM-based networks. Simulations with different threshold force criteria of spring rupture are shown using different symbols. Regression equations are also given next to the corresponding regression lines.
